# One-step synthesis of Fe_3_PtPd(OH)_2_[Picolinic acid]_8_(H_2_O)_4_ hybrid nanorods: efficient and stable electrocatalyst for oxygen reduction reaction in alkaline solution

**DOI:** 10.1038/s41598-018-33166-8

**Published:** 2018-10-17

**Authors:** Hamid Asiabi, Yadollah Yamini, Maryam Shamsayei, Esmaiel Saievar-Iranizad, Amir Bayat, Saeid Kamari kaverlavani

**Affiliations:** 1 Department of Chemistry, Tarbiat Modares University, P.O. Box 14115-175, Tehran, Islamic Republic of Iran; 2 Department of Physics, Faculty of Science, Tarbiat Modares University, Tehran, Islamic Republic of Iran

## Abstract

Design and synthesis of effective electrocatalysts for oxygen reduction reaction in alkaline environments is critical to reduce energy losses in alkaline fuel cells. We have systematically evaluated new approaches for reducing the Pt content while retaining the activity of a Pt-based catalyst with hydrolytic phases containing hydroxide moieties in addition to metal ions and ligands. We report for the first time architectured organic-inorganic hybrid nanorod catalyst, which is fabricated by solvothermal reaction of K_2_MCl_4_ (M = Pd, Pt) with picolinic acid (PA) (chelating agent) in the presence of FeCl_2_. Excess base produces isostructural coordination M-PA complex to Fe-OH chains. A generic formula can be written as Fe_3_PtPd(OH)_2_[PA]_8_(H_2_O)_4_. The electrocatalytic activities of the hybrid nanorods are explored for oxygen reduction reaction (ORR) in alkaline medium. The onset potential of ORR is significantly reduced with a positive shift of about 109 mV and twice the reduction current density is observed in comparison with Pt/C with the same mass loading. We believe that this work may lead towards the development of heterodoped organic- inorganic hybrid materials with greatly enhanced activity and durability for applications in catalysis and energy conversion.

## Introduction

Energy is one of the biggest challenges in the 21st century and there has been an ever increasing demand for environmental friendly high-power energy sources. Fuel cells with their high energy and power density have been widely considered as green and efficient alternative energy sources^1,2^. The cathodic oxygen reduction reaction (ORR) is at the heart of fuel cell performance, and efficient ORR electrocatalysts are essential for practical applications. The common commercial catalysts used in ORR are Pt and Pt based precious metal electrocatalysts, which exhibit excellent catalytic activity, but suffer from prohibitive cost, susceptibility to methanol crossover, and poor stability in the electrochemical environments^3–7^. Recent efforts in electrocatalysis have focused on decreasing the Pt content in fuel cell electrocatalysts or replacing it with less expensive materials^8–12^. In the face of these considerable challenges, one of the pathways to address these problems is to increase the ORR activity as well as reduce cost by alloying a second metallic element such as Pd, Fe, Ni, and Co with pure Pt^12–16^. These catalysts are more active for ORR than their Pt counterparts. For example, recent research has demonstrated that Pd doped Pt systems show excellent ORR performance in both alkaline and acidic electrolytes^17,18^. The cost of Pd, however, is currently about one-third that of Pt, and it is at least 50 times more abundant than Pt. In addition, for ORR, it has been found that a small amount of Fe doping is effective for enhancing the ORR activities of Pt or Pd alloys, possibly due to the enhanced structure disorder and conductivity^19,20^. For example, the unique core–shell structures of Pd/FePt NPs with 5 nm Pd core encircled with a FePt shell generate 12 times more current (5 nm/1 nm Pd/FePt NPs) than commercially available pure-Pt catalysts^19^. On the other hand, transition-metal species, such as metal hydroxides or layered double hydroxides, have recently gained noticeable popularity in various energy systems owing to their low cost and high theoretical activity^21^. For example, Lei *et al*. report on the highly active and durable Ni_x_Co_1−x_(OH)_2_ catalyst for ORR^22^. Li *et al*. observed an excellent ORR performance on NiCoFe-LDH^23^. These results demonstrate that 3D metal in the hydroxide state is a promising catalyst for ORR. These studies give the idea that the use of noble and non-noble metals together in the hydroxide or complex form, instead of the reduced form, can effectively increase ORR activity. But usually, heterogeneous electrocatalysts suffer from extensive leaching of the active metal species during reactions and eventually lose their catalytic activity.

Herein, for the first time, we report an architectured organic-inorganic hybrid nanorod electrocatalyst, which was fabricated by the solvothermal reaction of K_2_MCl_4_ (M = Pd, Pt) with picolinic acid in the presence of FeCl_2_ (in which hydroxide forms easily in the basic solution). The excess base produces isostructural coordination solids in which ‘complex ligands’, containing palladium or platinum, coordinate to metal hydroxide chains. A generic formula can be written as: Fe_3_M_2_(OH)_2_[PA]_8_(H_2_O)_4_, where M^2+^  = Pd and Pt^24^. PA is known to be stable in solvothermal conditions and also to form stable complexes with soft metals via -NH coordination and hard metals –OH coordination^25^. This synthesis method allows to form a catalyst with a uniform distribution of metal ions at structure. In these conditions, the Pt content of the catalyst decreases while the activity of a Pt-based catalyst with hydrolytic phases containing hydroxide moieties will be retained. Also, This structure decreases metal leaching in successive runs and increases reusability of the catalyst. To study the effect of the presence of Pt and Pd on ORR activity, three types of hybrid nanorods, including Fe_3_Pt_2_[PA]_8_(OH)_2_(H_2_O)_4_, Fe_3_Pd_2_[PA]_8_(OH)_2_(H_2_O)_4_ and Fe_3_PtPd[PA]_8_(OH)_2_(H_2_O)_4_, were synthesized, and the effect of their compositions on ORR activity was studied. These hybrid nanorods displayed substantially enhanced ORR activity as compared with that of commercial Pt/C catalysts in 0.1 M KOH solution. It is critical to highlight that, to the best of our knowledge, there have been no previous reports of either supported or activated Fe(OH)_2_ with Pt and Pd complexes. These hybrid nanorods are a promising new catalyst candidate for practical fuel cell applications.

## Results

### Characterization of hybrid nanorods

**Figure 1:**
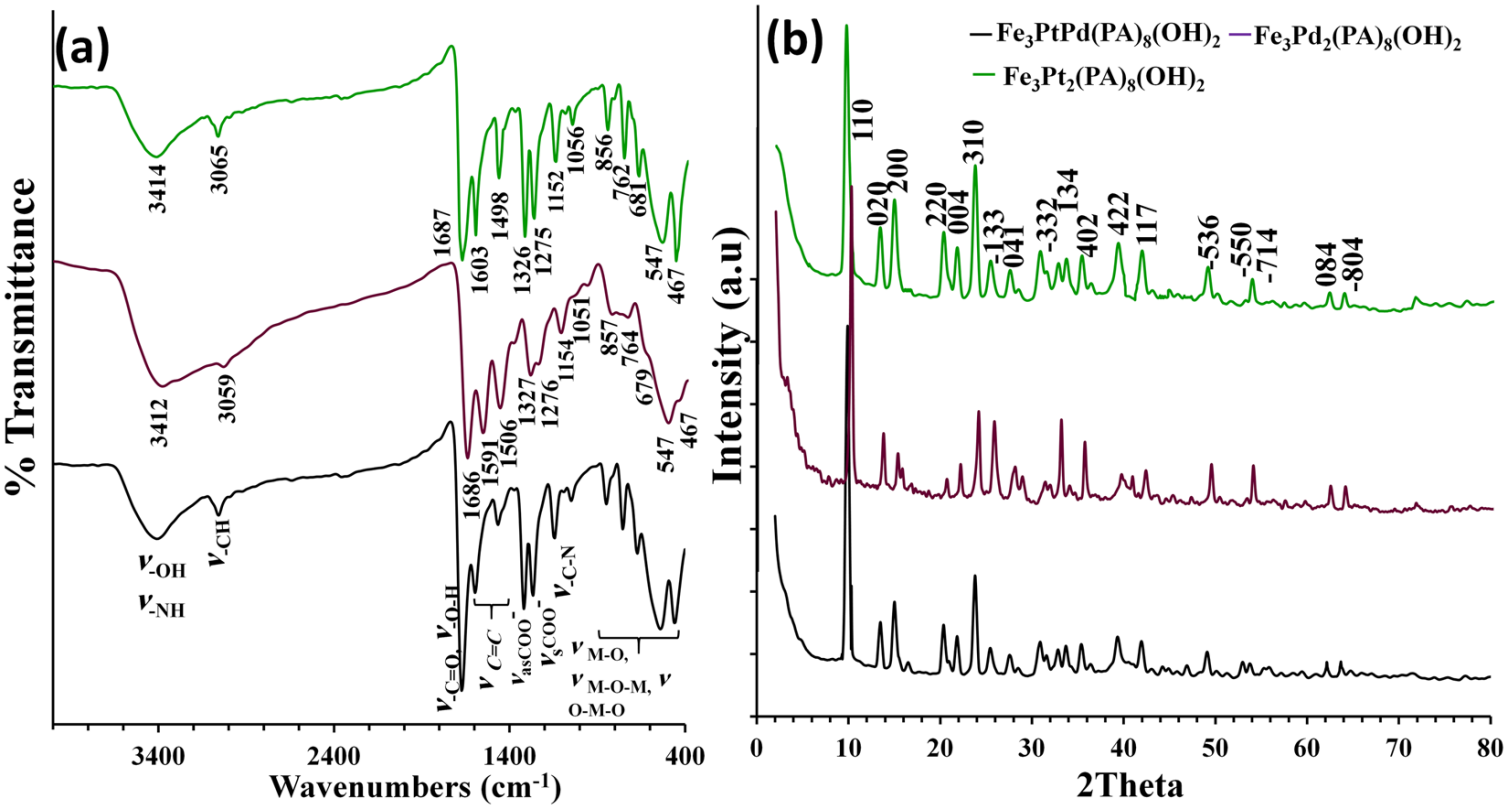
(**a**) FT-IR spectra, (**b**) XRD patterns of Fe_3_Pt_2_[PA]_8_(OH)_2_(H_2_O)_4_ (green lines), Fe_3_Pd_2_[PA]_8_(OH)_2_(H_2_O)_4_ (violet lines), and Fe_3_PtPd[PA]_8_(OH)_2_(H_2_O)_4_ (black lines).

FT-IR spectroscopy, X-ray diffraction (XRD), scanning electron microscopy (SEM), EDX, and transmission electron microscopy (TEM) were applied for characterization. FTIR spectra for the as-prepared nanohybrides are shown in Fig. 1a. The FTIR spectra of nanohybrides were roughly attributed to what follows: 3412 cm^−1^ to the O-H and N-H stretching vibration of the Fe hydroxide layer and PA amine groups; 3056 cm^−1^ due to the alkyl C-H stretching of PA; 1687 cm^−1^ due to the carboxylic acid C=O stretching of PA and the OH stretching band of H_2_O; 1506–1591 cm^−1^ due to the aromatic C=C bending; 1327 cm^−1^ due to the ν_-COO-_ (asymmetric); and 1276 cm^−1^ due to the ν_-COO-_ (symmetric); 1154 cm^−1^ due to the –C-N stretching; and 500–900 cm^−1^ to the Fe-O, Fe-O-Fe, and O-Fe-O lattice vibrations. These observations confirmed the formation of Pt/Pd-PA complex and Fe-hydroxide at all three hybrid nanorods.

Figure 1b illustrates the XRD pattern of hydrothermally synthesized hybrid nanorods. From Fig. 1b, it can be seen that the observed reflections clearly indicate the formation of a single phase compound without any impurity traces. XRD results revealed that all of three formed hybrid nanorods have a monoclinic crystal structure with a C2/c space group (JCPDS card no. 00-049-2426). The structure of monoclinic nanorod hybrids is composed of two different structures including metal hydroxide (C2/c space group and JCPDS card no. 00-030-0147) and M-PA complex with monoclinic structure (P21/c space group and JCPDS card no. 00-044-1812), which are held together by two different types of linkage. The first type of linkage consists of infinite chains with a –Fe/PA/M/PA/Fe/PA/M– repeat unit. The second type is a series of Fe hydroxide strips. Each individual layer has metal hydroxide ‘ribs’, which are linked to their nearest neighbors by a row of PA/M/PA moieties^24–27^.

**Figure 2:**
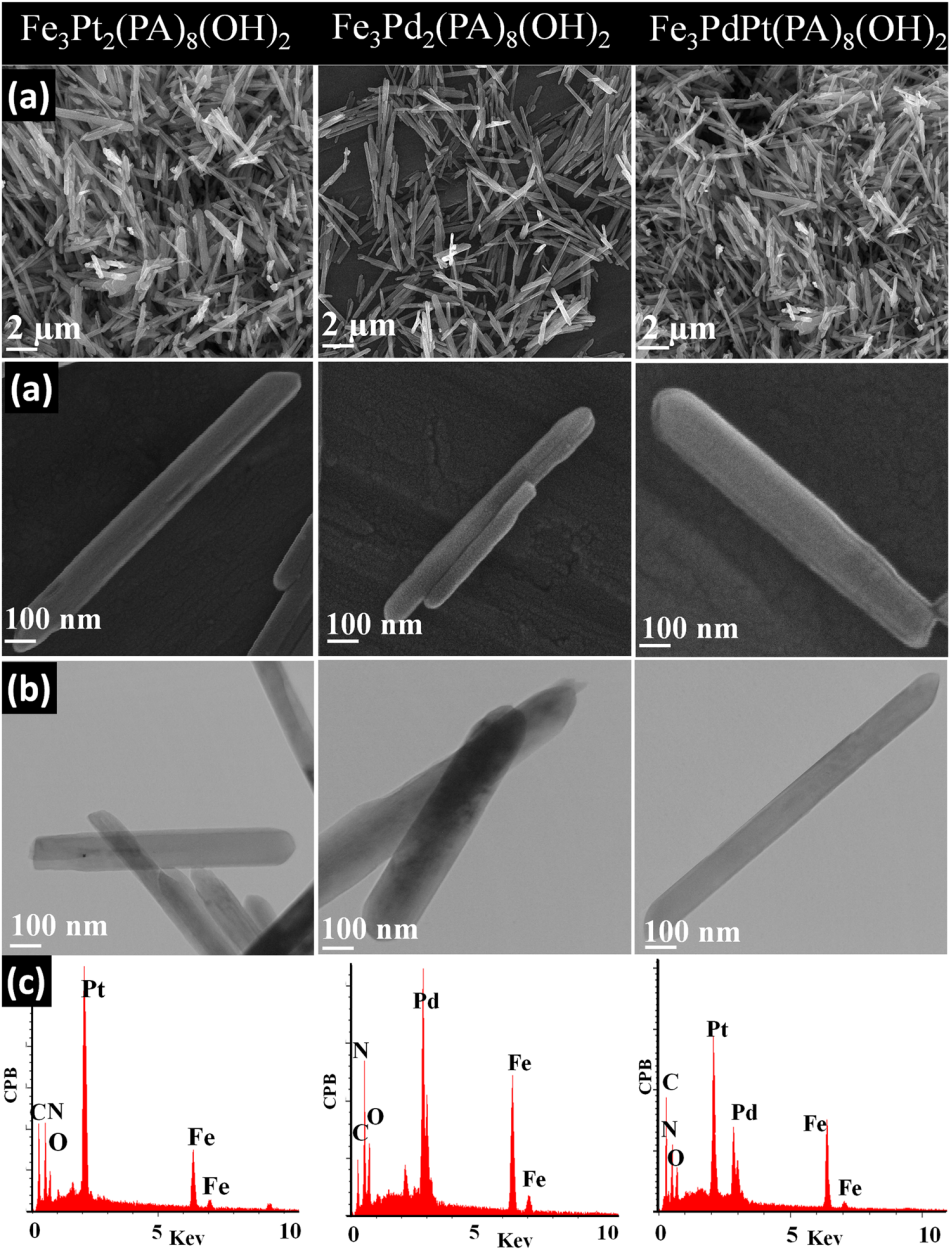
Structural and compositional analyses of the hybrid nanorods. (**a**) Low- and high- magnification FE-SEM images of the hybrid nanorods, (**b**) TEM images of the hybrid nanorods, (**c**) EDS spectrum of the hybrid nanorods.

The surface composition of the Fe_3_PtPd(OH)_2_[PA]_8_(H_2_O)_4_ was analyzed by X-ray photoelectron spectroscopy (XPS, Figure S1). The survey spectrum (Figure S1a) shows carbon, oxygen, nitrogen, iron, palladium and platinum species. The high-resolution XPS spectra of C 1 s (Figure S1b) showed that the C 1 s region contains five components corresponding to C=C, C–OH, C=O, HO-C=O and C=N species can be further separated out. The O 1 s region (Figure S1c) contains four components corresponding to Pt/Pd/Fe-OH, C=O, C-O, and O-C=O bonds. The N1s, Pd 3d and Pt 4 f XPS spectra are shown in Fig. 2d–f, respectively. The increase in the binding energy of element N and decrease in the binding energies of element Pd and Pt was occurred due to the fitting the surplus pair electrons of element N in the free electron orbital of element Pd and Pt in the chelating process. The positive shifts of the N 1 s peak and negative shifts of the Pd 3d and Pt 4 f peaks suggest that the intermolecular coordination of palladium/platinum with nitrogen of the picolinic acid happened. The shift of binding energy could affect the interaction strength between Pd (II)/Pt (II) ions and picolinic acid. In Figure S1g, the XPS spectrum of the Fe shows double peaks with binding energies at 706.3 eV and 719.8 eV, corresponding to Fe 2p_3/2_ and Fe 2p_1/2_, respectively. Compared to the standard spectra of metal Fe(II), the binding energies of Fe decreases slightly. These results combined with obtained results from O 1 s spectra confirm the Fe(OH)_2_ formation. XPS was used to confirm the valence state of the Pd, Pt and Fe on the catalyst. As shown in Fig. 2e–g, the Pd 3d, Pt 4 f and Fe 2p XPS spectra of catalyst are attributed to Pd(II), Pt(II) and Fe(II).

Morphological properties of all three hybrid nanorods were analyzed by FE-SEM and TEM, and the images are shown in Fig. 2. From FE-SEM and TEM images, it is apparent to notice the formation of the regular shaped hybrid nanorods with ~100 nm thickness and 2–3 µm length for all three hybrid nanorods (Fig. 2a,b). The elemental analysis of the as-prepared hybrid nanorods was obtained using EDX and ICP-AES. According to Fig. 2c, the formulas of the hybrid nanorods are derived as Fe_3_Pt_2_[PA]_8_(OH)_2_(H_2_O)_4_, Fe_3_Pd_2_[PA]_8_(OH)_2_(H_2_O)_4_, and Fe_3_PtPd[PA]_8_(OH)_2_(H_2_O)_4_, which is strong evidence for the formation of the hybrid nanorods. SEM-EDX mapping analysis for the hybrid nanorods (Figure S2) proved the uniform distribution of Pt, Pd, Fe and C, N, O (Picolinic acid) inside structure. The uniform structure lead to enhancement in both catalytic activity and durability toward the oxygen reduction reaction.

### Activity and performance of hybrid nanorods for the ORR

**Figure 3:**
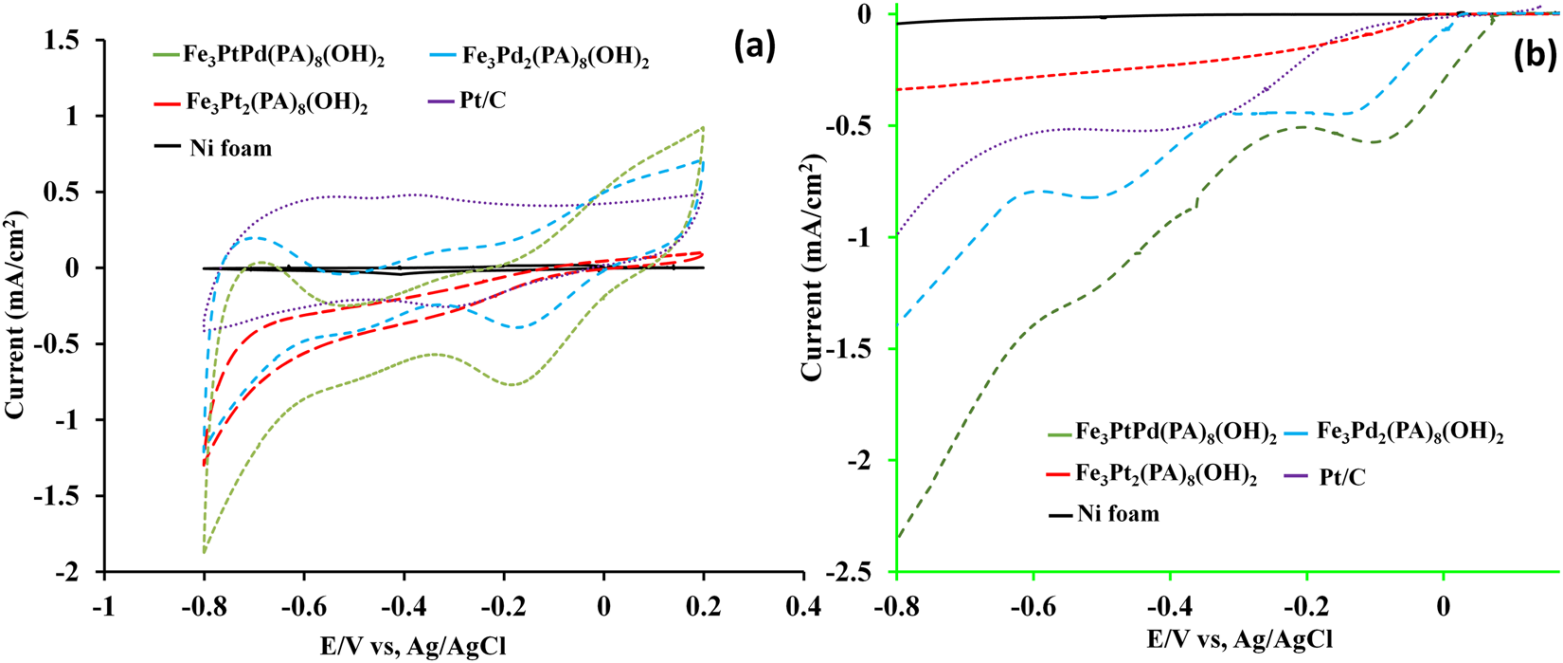
(**a**) Cyclic voltammograms of oxygen reduction reaction on Pt/C, Ni foam, Fe_3_Pt_2_[PA]_8_(OH)_2_(H_2_O)_4_, Fe_3_Pd_2_[PA]_8_(OH)_2_(H_2_O)_4_, and Fe_3_PtPd[PA]_8_(OH)_2_(H_2_O)_4_ in a O_2_-saturated 0.1 M KOH solution. Scan rate = 10 mV s^−1^; room temperature. (**b**) LSV polarization curves for Pt/C, Ni foam, Fe_3_Pt_2_[PA]_8_(OH)_2_(H_2_O)_4_, Fe_3_Pd_2_[PA]_8_(OH)_2_(H_2_O)_4_, and Fe_3_PtPd[PA]_8_(OH)_2_(H_2_O)_4_ in a O_2_-saturated 0.1 M KOH solution. Scan rate = 10 mV s^−1^; room temperature.

We first compared the electrochemical behavior of the hybrid nanorods with each other and with Pt/C for ORR using cyclic voltammograms (CVs) cell in oxygen-saturated 0.1 M KOH (Fig. 3a). The detailed information can be found in Table 1. As can be seen in Fig. 3a, distinct peaks corresponding to ORRs can be observed for all the hybrid nanorods. In Fig. 3a, single cathodic reduction peaks at −0.3 V, −0.18 V, and −0.09 V can be observed in an O_2_-saturated solution for the Fe_3_Pt_2_[PA]_8_(OH)_2_(H_2_O)_4_, Fe_3_Pd_2_[PA]_8_(OH)_2_(H_2_O)_4_, and Fe_3_PtPd[PA]_8_(OH)_2_(H_2_O)_4_ electrodes, respectively. Most interesting is that in the O_2_-saturated solution, ORR peak potential of the Pd based hybrid nanorods is more positive than that of Pt based hybrid nanorods. Additionally, compared with Fe_3_Pt_2_[PA]_8_(OH)_2_(H_2_O)_4_, Fe_3_Pd_2_[PA]_8_(OH)_2_(H_2_O)_4_, a significant increase of the peak current density and an obvious positive shift of the peak potential can be found on Fe_3_PtPd[PA]_8_(OH)_2_ (H_2_O)_4_. Commercial Pt/C catalysts exhibit a reduction peak at −0.27 V for ORR. Obtained results showed that the peak potential for reduction of oxygen at the hybrid nanorods, especially Fe_3_PtPd[PA]_8_(OH)_2_(H_2_O)_4_, is more positive than that of commercial Pt/C, shifting positively by about 0.18 mV. The higher peak current density and more positive reduction potential observed in CVs suggests that Fe_3_PtPd[PA]_8_(OH)_2_(H_2_O)_4_ catalysts exhibit much better catalytic activity toward ORR.

**Table 1:** Summary of the important performance parameters of the ORR catalysts extracted from the LSV results shown in Fig. 3.

Catalyst	Onset potential	Current density (mA/cm^2^) at −0.8 V	Half-wave potential
Fe_3_Pt_2_(PA)_8_(OH)_2_	−0.014	−0.34	−0.3
Fe_3_Pd_2_(PA)_8_(OH)_2_	0.024	−1.38	−0.18
Fe_3_PdPt(PA)_8_(OH)_2_	0.087	−2.36	−0.09
Pt/C	−0.022	−0.99	−0.27

LSVs in O_2_-saturated 0.1 M KOH (Fig. 3b) were conducted to further investigate the ORR activity of Fe_3_Pt_2_[PA]_8_(OH)_2_(H_2_O)_4_, Fe_3_Pd_2_[PA]_8_(OH)_2_(H_2_O)_4_, and Fe_3_PtPd[PA]_8_(OH)_2_(H_2_O)_4,_ and then they were compared with the commercial Pt/C catalyst. The catalytic parameters for the ORR are summarized in Table 1. The obtained results from Fig. 3b demonstrate that the ORR process catalyzed by the Fe_3_PtPd[PA]_8_(OH)_2_(H_2_O)_4_ and Fe_3_Pd_2_[PA]_8_(OH)_2_(H_2_O)_4_ are a two-step two-electron pathway with the formation of intermediate HO_2_^−^ ions, consistent with the reports in the literature^28,29^. By contrast, Fe_3_Pt_2_[PA]_8_(OH)_2_(H_2_O)_4_ exhibits a one-step process for ORR. As Fig. 3b shows, the onset potentials measured for all the hybrid nanorods are positively moved from that of Pt/C. The observed onset potentials order for the hybrid nanorods and Pt/C was Fe_3_PtPd[PA]_8_(OH)_2_(H_2_O)_4_ (E_onset_ = 0.087 V) > Fe_3_Pd_2_[PA]_8_(OH)_2_(H_2_O)_4_ (E_onset_ = 0.024 V) > Fe_3_Pt_2_[PA]_8_(OH)_2_(H_2_O)_4_ (E_onset_ = −0.014 V) > Pt/C (E_onset_ = −0.022 V). It is clear that the ORR onset potential measured for all the hybrid nanorods are positively moved from that of Pt/C. These results indicate that ORR activity of the Pd based hybrid nanorods is better than that of the Pt based hybrid nanorod, which is in line with previous reports^30,31^. However, the Fe_3_PtPd[PA]_8_(OH)_2_(H_2_O)_4_ catalyst displays the most positive onset potential and the largest limiting current density compared with Fe_3_Pt_2_[PA]_8_(OH)_2_(H_2_O)_4_ and Fe_3_Pd_2_[PA]_8_(OH)_2_(H_2_O)_4_ catalysts. The limiting current density on Fe_3_PtPd[PA]_8_(OH)_2_(H_2_O)_4_ is nearly twice and seven times as large as that on Fe_3_Pd_2_[PA]_8_(OH)_2_(H_2_O)_4_ and Fe_3_Pt_2_[PA]_8_(OH)_2_(H_2_O)_4_, respectively. Furthermore, the Fe_3_PtPd[PA]_8_(OH)_2_ (H_2_O)_4_ catalyst exhibits considerable catalytic performance with better onset potential, half-wave potential, and reduction current compared to the commercial Pt/C. The onset potential of Fe_3_PtPd[PA]_8_(OH)_2_(H_2_O)_4_ is more positive than that of Pt/C, shifting positively by about 109 mV. The enhanced specific activity for the Fe_3_PtPd[PA]_8_(OH)_2_(H_2_O)_4_ catalyst should be attributed to the strain arising from the mismatch in the lattice constant among Pd, Pt, and Fe, and the ligand effect reflecting the electronic coupling among the three metals^3,32–35^. These results suggest that the ORR catalytic activity of Fe_3_PtPd[PA]_8_(OH)_2_(H_2_O)_4_ is better than that of commercial Pt/C catalysts, Fe_3_Pt_2_[PA]_8_(OH)_2_(H_2_O)_4_ and Fe_3_Pd_2_[PA]_8_(OH)_2_(H_2_O)_4_, indicating the advanced role of doped heteroatoms to enhance ORR activity.

**Figure 4:**
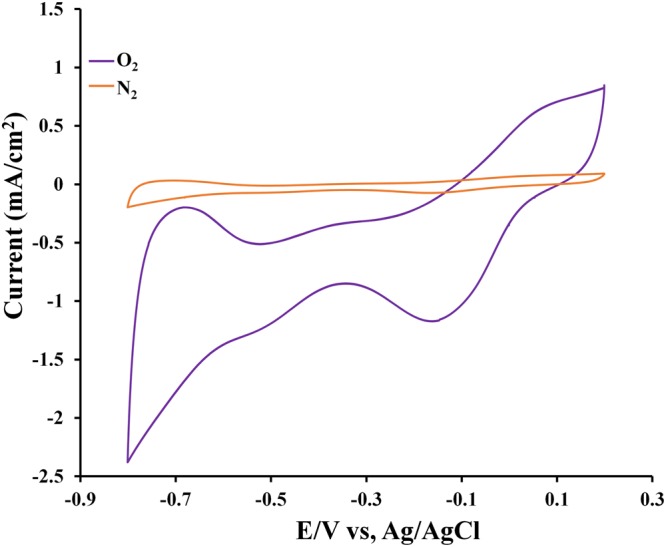
The CV curves of Fe_3_PtPd[PA]_8_(OH)_2_(H_2_O)_4_ in O_2_ saturated (violet line) and N_2_ saturated (orange line) in 0.1 M KOH solution; scan rate = 10 mV s^−1^; room temperature.

To demonstrate the practical electrocatalytic activity of Fe_3_PtPd[PA]_8_(OH)_2_(H_2_O)_4_ towards oxygen reduction, the CV of the electrode in 0.1 M KOH solutions saturated with N_2_ or O_2_ was investigated at a scan rate of 10 mV s^−1^. As shown in Fig. 4, the CV curve of Fe_3_PtPd[PA]_8_(OH)_2_(H_2_O)_4_ obtained from the N_2_-saturated solutions show featureless voltammetric currents in the range of 0.2 to −0.8 V, while the CV curve obtained from the O_2_ saturated solution exhibit well-defined cathodic peaks corresponding to the reduction of oxygen. This clearly demonstrates the electrocatalytic activity of Fe_3_PtPd[PA]_8_(OH)_2_(H_2_O)_4_ towards oxygen reduction.

### Stability studies

**Figure 5:**
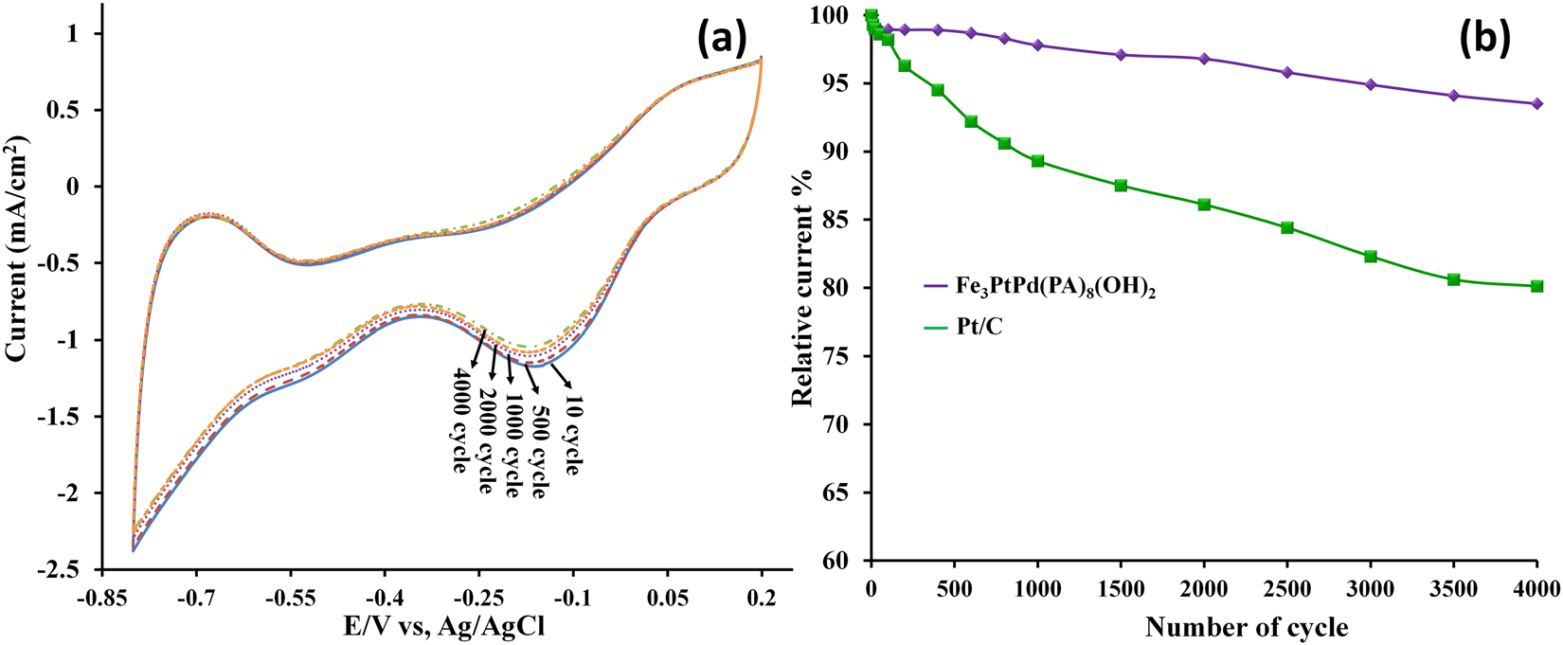
(**a**) Stability studies of Fe_3_PtPd[PA]_8_(OH)_2_(H_2_O)_4_ with continuous CV cycles in 0.1 M KOH solution saturated with oxygen: the sweep rate is 10 mV s^−1^. The dotted line shows the difference from the 10st cycle to the 4000th cycle. (**b**) The comparison of the ORR stability of Fe_3_PtPd[PA]_8_(OH)_2_(H_2_O)_4_ with that of the commercial Pt/C in O_2_ saturated 0.1 M KOH from the initial cycle to the 4000th cycle.

The durability of the catalysts and the long-term stability of the electrocatalytic activity for ORR are of prominent concern in fuel cells. The stabilities of the Fe_3_PtPd[PA]_8_(OH)_2_(H_2_O)_4_ and Pt/C electrodes towards oxygen reduction are shown in Fig. 5. The dotted lines are the cycling difference from the 1st cycle to the 4000th cycle. Pt/C catalysts display a rapid decay of the signal (up to 20%) current depression after the 4000th cycle, indicating a poor stability. In contrast, the response of the Fe_3_PtPd[PA]_8_(OH)_2_(H_2_O)_4_ electrode retains acceptable stability throughout the entire experiment, with only 5% current diminutions after the 4000th cycle. These results demonstrate the higher durability of Fe_3_PtPd[PA]_8_(OH)_2_(H_2_O)_4_ compared with Pt/C. The promoted electrochemical stability may be due to the stronger interaction force between Pt/Pd-PA and F-OH groups than the force between Pt and C.

### Scan rate effect

**Figure 6:**
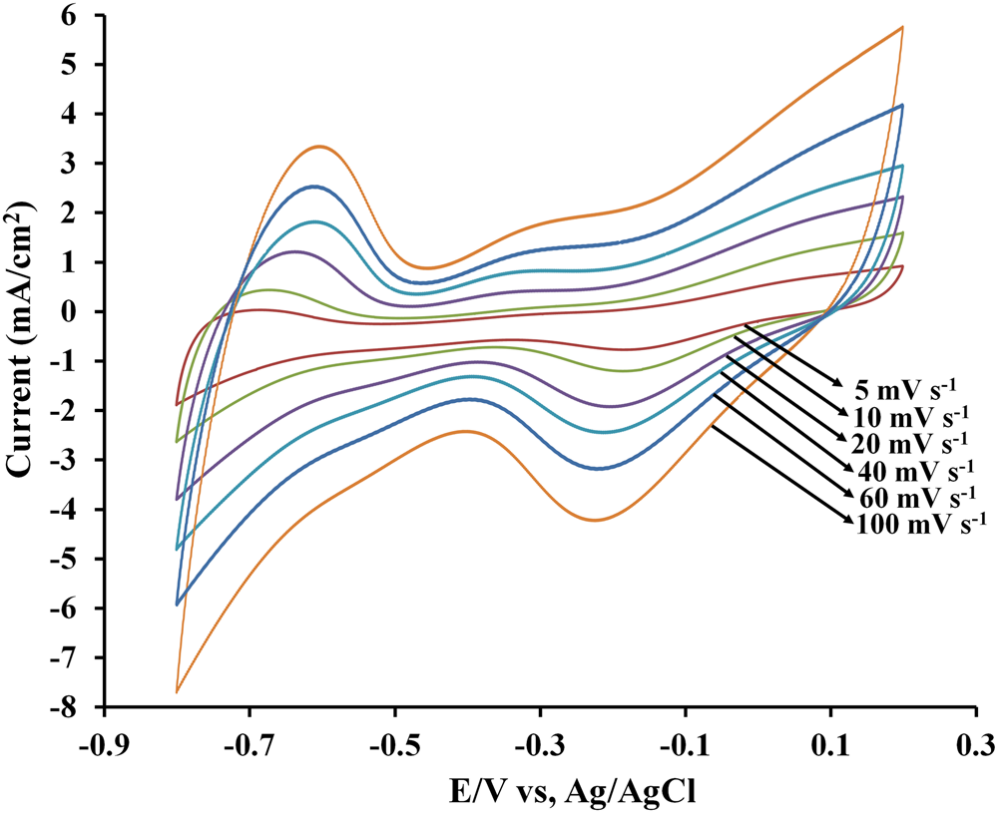
CV curves for oxygen reduction on a Fe_3_PtPd[PA]_8_(OH)_2_(H_2_O)_4_ electrode in O_2_-saturated 0.1 M KOH at different scan rates. *v* = 5–100 mV s^−1^.

Figure 6 shows the representative CV curves of the Fe_3_PtPd[PA]_8_(OH)_2_(H_2_O)_4_ electrode in a 0.1 M KOH aqueous electrolyte at various scan rates ranging from 5 to 100 mV s^−1^. Clearly, an ORR peak within 0.1 to −0.4 V is visible in all the CV curves. Furthermore, a linear relation between the peak current at different scan rates and the square root of the scan rate is observed, confirming that the redox reaction is a diffusion-controlled process.

These results evidently suggest that the great promise of this hybrid Ni foam supported the Fe_3_PtPd[PA]_8_(OH)_2_(H_2_O)_4_ nanorods electrode for high-performance ORR by long cycle life, excellent onset potential, half-wave potential, and reduction current.

## Discussions

In summary, we have fabricated an advanced organic-inorganic hybrid nanorod catalyst with the generic formula of Fe_3_PtPd[PA]_8_(OH)_2_ (H_2_O)_4_, which boasts a greater electrocatalytic ORR activity than that of current commercial Pt/C catalysts. Specifically, the enhancement in the specific activity can be attributed to a uniform distribution of Pt and Pd complexes at Fe-OH structure, ligand effect, and strain effect arising from the lattice mismatch between Pd, Pt, and Fe. The new types of heterodoped structures may provide opportunities for further development of catalysts with high activities and a long lifetime for practical ORR applications in alkaline solutions.

## Materials

### Synthesis of hybrid nanorods

Hybrid nanorods with a molar ratio of Pd:Pt from 1:0 to 0:1 were prepared by solvothermal treatment. A similar synthesis method was previously reported^24^. Briefly, aqueous solution A (0.1 mol K_2_MCl_4_ (M = Pt, Pd with the molar ratio of 0:1, 1:1 and 1:0) in 25 mL of CO_2_-free deionized water), solution B (picolinic acid 0.2 mol in 25 mL of CO_2_-free deionized water), and solution C (FeCl_2_ 0.2 mol in in 25 mL of CO_2_-free deionized water) were prepared. Solutions (A) and (B) were first mixed, and the pH of the solution was adjusted to 10.0 by addition of 2 M of NaOH solution. Then, solution C was added dropwise with stirring. The solution pH was controlled in the range of 9.5–10.5. The mixture was placed in a 100 mL Teflon-lined stainless autoclave and heated at 150 °C for 15 h. After being cooled to room temperature, the obtained precipitate was filtered and washed with a large amount of water until the pH value of the waste water reached 7, and then it was dried at 70 °C for 6 h.

### Oxygen reduction reaction procedure

Electrochemical measurements for evaluation of ORR catalytic activity of the hybrid nanorods were performed using a computer-controlled potentiostat (CHI 760 C, CH Instrument, USA) with a typical three-electrode system. A nickel foam electrode (0.3 mm diameter) was used as the working electrode, a Pt foil as the counter electrode, and a saturated Ag/AgCl electrode as the reference electrode. All the experiments were conducted at room temperature (25 °C). For working electrode preparation, 1.5 mg of the catalyst was dispersed in a mixture of 0.5 mL ethanol and 20 μL of 5% Nafion under ultrasonication for 20 min. Next, 10 μL of the dispersion was uniformly dropped onto the nickel foam electrode and dried at room temperature and under ambient conditions. The commercial Pt/C (20 wt% Pt on Vulcan XC-72) electrode was prepared by the same procedure.

## Electronic supplementary material

Supplementary Information

## References

[1] Yang Z (2011). Sulfur-doped graphene as an efficient metal-free cathode catalyst for oxygen reduction. ACS Nano.

[2] Zhu Y, Zhang B, Liu X, Wang DW, Su DS (2014). Unravelling the structure of electrocatalytically active Fe–N complexes in carbon for the oxygen reduction reaction. Angew. Chem. Int. Ed..

[3] Wang X (2015). Pd@ Pt core–shell concave decahedra: a class of catalysts for the oxygen reduction reaction with enhanced activity and durability. J. Am. Chem. Soc..

[4] Jiang Z, Jiang Z-j, Tian X, Chen W (2014). Amine-functionalized holey graphene as a highly active metal-free catalyst for the oxygen reduction reaction. J. Mater. Chem. A.

[5] Xiao L, Zhuang L, Liu Y, Lu J (2008). Activating Pd by morphology tailoring for oxygen reduction. J. Am. Chem. Soc..

[6] Morozan A, Jousselme B, Palacin S (2011). Low-platinum and platinum-free catalysts for the oxygen reduction reaction at fuel cell cathodes. Energy Environ. Sci..

[7] Zhang P (2014). ZIF-derived *in situ* nitrogen-doped porous carbons as efficient metal-free electrocatalysts for oxygen reduction reaction. Energy Environ. Sci..

[8] Markovic N, Schmidt T, Stamenkovic V, Ross P (2001). Oxygen reduction reaction on Pt and Pt bimetallic surfaces: a selective review. FUEL CELLS-WEINHEIM.

[9] Chai G-L (2017). Active sites engineering leads to exceptional ORR and OER bifunctionality in P, N Co-doped graphene frameworks. Energy Environ. Sci..

[10] Choi J-Y (2017). Is the rapid initial performance loss of Fe/N/C non precious metal catalysts due to micropore flooding?. Energy Environ. Sci..

[11] Lions M (2017). Insights into the mechanism of electrocatalysis of oxygen reduction reaction by a porphyrinic metal organic framework. Chem. Comm..

[12] Naveen Malenahalli Halappa, Shim Kyubin, Hossain Md. Shahriar A., Kim Jung Ho, Shim Yoon-Bo (2016). Template Free Preparation of Heteroatoms Doped Carbon Spheres with Trace Fe for Efficient Oxygen Reduction Reaction and Supercapacitor. Advanced Energy Materials.

[13] Zhu H, Zhang S, Huang Y-X, Wu L, Sun S (2013). Monodisperse M x Fe3–x O4 (M = Fe, Cu, Co, Mn) Nanoparticles and Their Electrocatalysis for Oxygen Reduction Reaction. Nano Lett..

[14] El-Deab MS, Sotomura T, Ohsaka T (2005). Oxygen reduction at electrochemically deposited crystallographically oriented Au (100)-like gold nanoparticles. Electrochem. Comm..

[15] Thorum MS, Hankett JM, Gewirth AA (2011). Poisoning the oxygen reduction reaction on carbon-supported Fe and Cu electrocatalysts: evidence for metal-centered activity. J. Phys. Chem. Lett..

[16] Hu, E. *et al*. Graphene Layers‐Wrapped Fe/Fe5C2 Nanoparticles Supported on N‐doped Graphene Nanosheets for Highly Efficient Oxygen Reduction. *Adv*. *Energy Mater*. (2017).

[17] Koenigsmann C (2011). Enhanced electrocatalytic performance of processed, ultrathin, supported Pd–Pt core–shell nanowire catalysts for the oxygen reduction reaction. J. Am. Chem. Soc..

[18] Fu G (2013). One-pot water-based synthesis of Pt–Pd alloy nanoflowers and their superior electrocatalytic activity for the oxygen reduction reaction and remarkable methanol-tolerant ability in acid media. J. Phys. Chem. C.

[19] Mazumder V, Chi M, More KL, Sun S (2010). Core/shell Pd/FePt nanoparticles as an active and durable catalyst for the oxygen reduction reaction. J. Am. Chem. Soc..

[20] Wakisaka M, Suzuki H, Mitsui S, Uchida H, Watanabe M (2008). Increased oxygen coverage at Pt− Fe alloy cathode for the enhanced oxygen reduction reaction studied by EC− XPS. J. Phys. Chem. C.

[21] Chen G (2014). Interfacial effects in iron-nickel hydroxide–platinum nanoparticles enhance catalytic oxidation. Science.

[22] Wang L (2014). A Comparative Study of Composition and Morphology Effect of Ni x Co1–x (OH) 2 on OxygenEvolution/Reduction Reaction. ACS Appl. Mater. Interfaces.

[23] Qian Li, Lu Zhiyi, Xu Tianhao, Wu Xiaochao, Tian Yang, Li Yaping, Huo Ziyang, Sun Xiaoming, Duan Xue (2015). Trinary Layered Double Hydroxides as High-Performance Bifunctional Materials for Oxygen Electrocatalysis. Advanced Energy Materials.

[24] Gerrard, L. A. & Wood, P. T. Hydrothermal crystal engineering using hard and soft acids and bases: synthesis and X-ray crystal structures of the metal hydroxide-based phases M3M′ 2 (OH) 2 [NC5H3 (CO2) 2-2, 4] 4 (H2O) 4 (M = Co, Ni, Zn; M′ = Pd, Pt). *Chem*. *Comm*. 2107–2108 (2000).

[25] Jayasree S, Seayad A, Chaudhari R (2000). Novel Palladium (II) Complex Containing a Chelating Anionic N−O Ligand: Efficient Carbonylation Catalyst. Org. Lett..

[26] Song R, Kim KM, Sohn YS (1999). Synthesis and properties of (diamine)platinum(II) complexes of pyridine carboxylate isomers and their antitumor activity. Inorg. Chim. Acta.

[27] Gong M (2013). An advanced Ni−Fe layered double hydroxide electrocatalyst for water oxidation. J. Am. Chem. Soc..

[28] Wang S (2012). BCN graphene as efficient metal‐free electrocatalyst for the oxygen reduction reaction. Angew. Chem. Int. Ed..

[29] Wang X (2014). One-pot synthesis of nitrogen and sulfur co-doped graphene as efficient metal-free electrocatalysts for the oxygen reduction reaction. Chem. Comm..

[30] Sha Y, Yu TH, Merinov BV, Shirvanian P, Goddard WA (2011). Oxygen hydration mechanism for the oxygen reduction reaction at Pt and Pd fuel cell catalysts. J. Phys. Chem. Lett..

[31] Jiang L, Hsu A, Chu D, Chen R (2009). Oxygen reduction reaction on carbon supported Pt and Pd in alkaline solutions. J. Electrochem. Soc..

[32] Xie S (2014). Atomic layer-by-layer deposition of Pt on Pd nanocubes for catalysts with enhanced activity and durability toward oxygen reduction. Nano Lett..

[33] Park J (2015). Atomic layer-by-layer deposition of platinum on palladium octahedra for enhanced catalysts toward the oxygen reduction reaction. ACS Nano.

[34] Wang, X. *et al*. Palladium-platinum core-shell icosahedra with substantially enhanced activity and durability towards oxygen reduction. *Nat*. *Comm*. **6** (2015). 10.1038/ncomms8594 PMC4506534 26133469

[35] Kitchin JR, Nørskov JK, Barteau MA, Chen J (2004). Role of strain and ligand effects in the modification of the electronic and chemical properties of bimetallic surfaces. Phys. Rev. Lett..

