# Supplementary material for: One-step synthesis of Fe3PtPd(OH)2[Picolinic acid]8(H2O)4 hybrid nanorods: efficient and stable electrocatalyst for oxygen reduction reaction in alkaline solution
Source: Sci Rep. 2018 Oct 17;8:15325. doi: 10.1038/s41598-018-33166-8 (PMC6193002; doi:10.1038/s41598-018-33166-8)
Supplement: Supplementary file 1 — Supplementary Information [file 41598_2018_33166_MOESM1_ESM.docx]

**Supporting information**

**One-step synthesis of Fe_3_PtPd(OH)_2_[Picolinic acid]_8_(H_2_O)_4_ hybrid nanorods: efficient and stable electrocatalyst for oxygen reduction reaction in alkaline solution**

Hamid Asiabi^a^, Yadollah Yamini^^[[1]](#footnote-1)^*,a^, Maryam Shamsayei^a^, Esmaiel Saievar-Iranizad^b^, Amir Bayat^b^, Saeid Kamari kaverlavani^b^

*^a^Department of Chemistry, Tarbiat Modares University, P.O. Box 14115-175, Tehran, Islamic Republic of Iran*

*^b^Department of Physics, Faculty of Science, Tarbiat Modares University, Tehran, Islamic Republic of Iran*

**Totals:** 5 pages, 1 table and 1 figure.

**Table of contents Page**

**Reagents and materials S3**

**Apparatus S3**

**Table S1 S4**

**Figure S1 S5**

**Figure S2 S6**

**Table S2 S6**

**Figure S3 S7**

**Reagents and materials**

All chemicals were of analytical reagent grade. FeCl_2_, K_2_PtCl_4_ and K_2_pPdCl_4_ salts and picolinic acid were purchased from Sigma-Aldrich (Milwaukee, WI, USA). The water consumed was purified on a Youngling ultrapure water purification system model Aqua MaxTM-ultra (Seoul, South Korea). Other chemicals applied were of analytical reagent grade or of the highest purity available.

**Apparatus**

Particle size and morphology of the synthesized nanoparticles were determined by a scanning electron microscope (SEM) model EM3200 from KYKY Zhongguancun (Beijing, China). In order to structural study of the catalysts, XRD measurements were performed on a Philips (Netherland, Holland) X-ray powder diffractometer (Cu Kα radiation source, λ = 0.154056 nm) between 1^◦^ to 80^◦^ generated at 40 kV and 30 mA at room temperature. Test angle of 1–10° with a scan rate of 1°/min and 10–80° with a scan rate of 5°/min were employed, respectively. Samples for XRD were ground into powder and then pressed flat in the sample slot. In addition, FT-IR spectra (4000–400cm^−1^) were recorded on a thermo Scientific Nicolet IR100 (Madison, WI, USA) Fourier-transform infrared (FTIR) spectrometer using the KBr disk method with a ratio sample/KBr of 1:100 by mass. UV–vis absorption spectra were measured using a Model Scinco UV S-2100. Fluorescence spectra were obtained using a Perkin-Elmer LS-50B fluorescence spectrometer equipped with a xenon flash lamp. Transmission electron microscopy (TEM) images were taken on a Zeiss transmission electron microscope operating at an accelerating voltage of 150 kV. Simultaneous inductively coupled plasma atomic emission spectrometry (ICP-AES) on a Varian Vista-PRO instrument (Springvale, Australia) with a radial torch coupled to a concentric nebulizer and a Scott spray chamber and equipped with a charge-coupled detector (CCD) was used for simultaneous determination of the target elements. A six-port two-position injection valve from Tehran University (Tehran, Iran) equipped with a 250 μL injection loop constructed from silicon tubing was used to introduce the final solution into the ICP-AES nebulizer. Table S1 lists the optimal instrumental conditions and the emission lines employed for the determination of the metal ions by ICP-AES.

| Table S1. ICP-AES operating conditions and metal ions emission lines. | |
| --- | --- |
| Plasma gas | Argon |
| Plasma gas flow rate | 15 L min^-1^ |
| Auxiliary gas flow rate | 1.5 L min^-1^ |
| Frequency of RF generator | 40 MHz |
| RF generator power | 1.2 kW |
| Observation height | 8 mm |
| Nebulizer pressure | 180 kPa |
| Eluent | Deionized water |
| Elution rate | 2 mL min^-1^ |
| Analytical lines | Fe (238.204), Pt (265.945), Pd (283.305), |


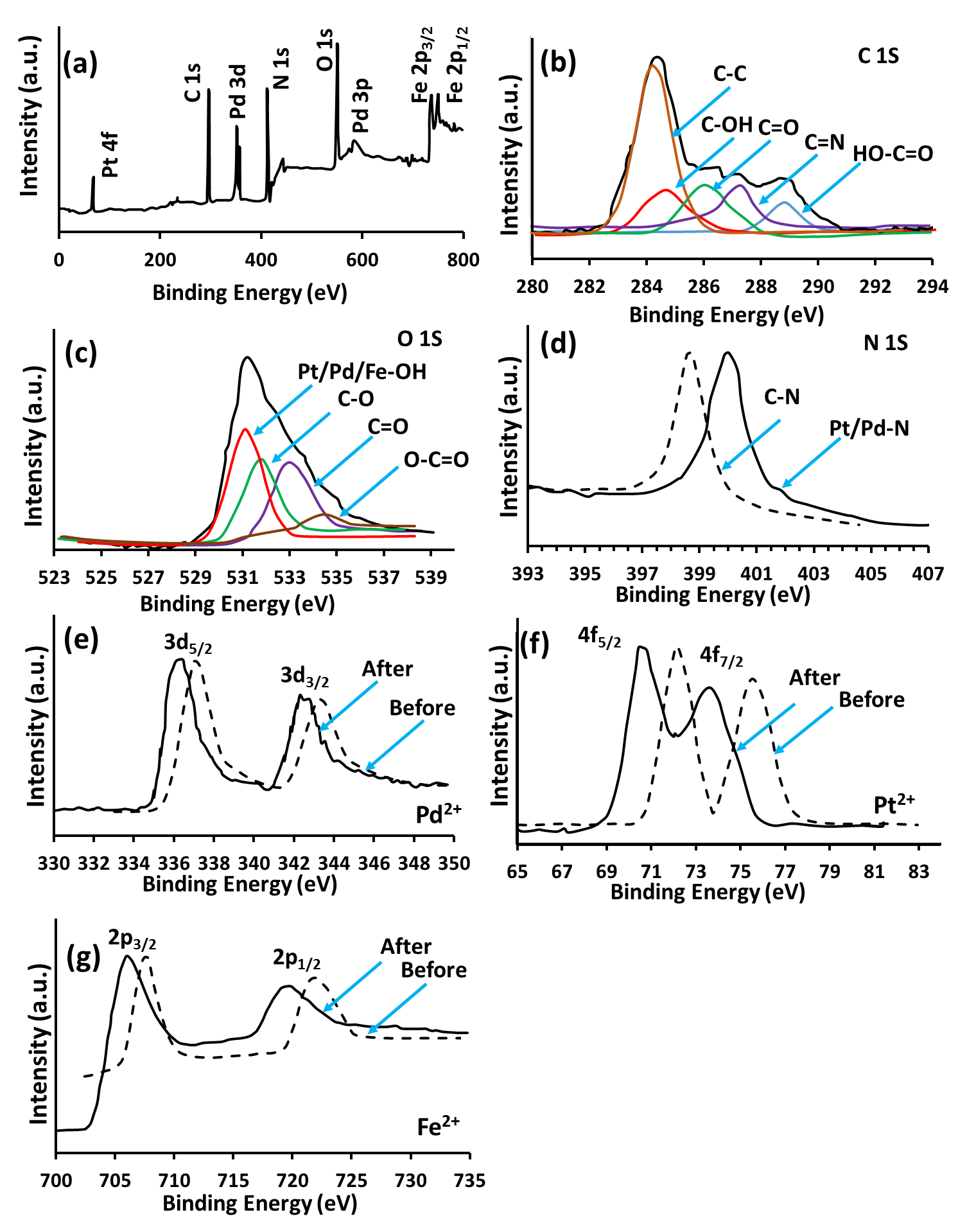


Figure S1 XPS survey spectrum of Fe3PtPd (OH)_2_[PA]_8_(H_2_O)_4_ (a) and enlarged spectra in C 1s (b), O 1s (c), N 1s (d), Pd 3d (e), Pt 4f, and Fe 3p (g) regions.


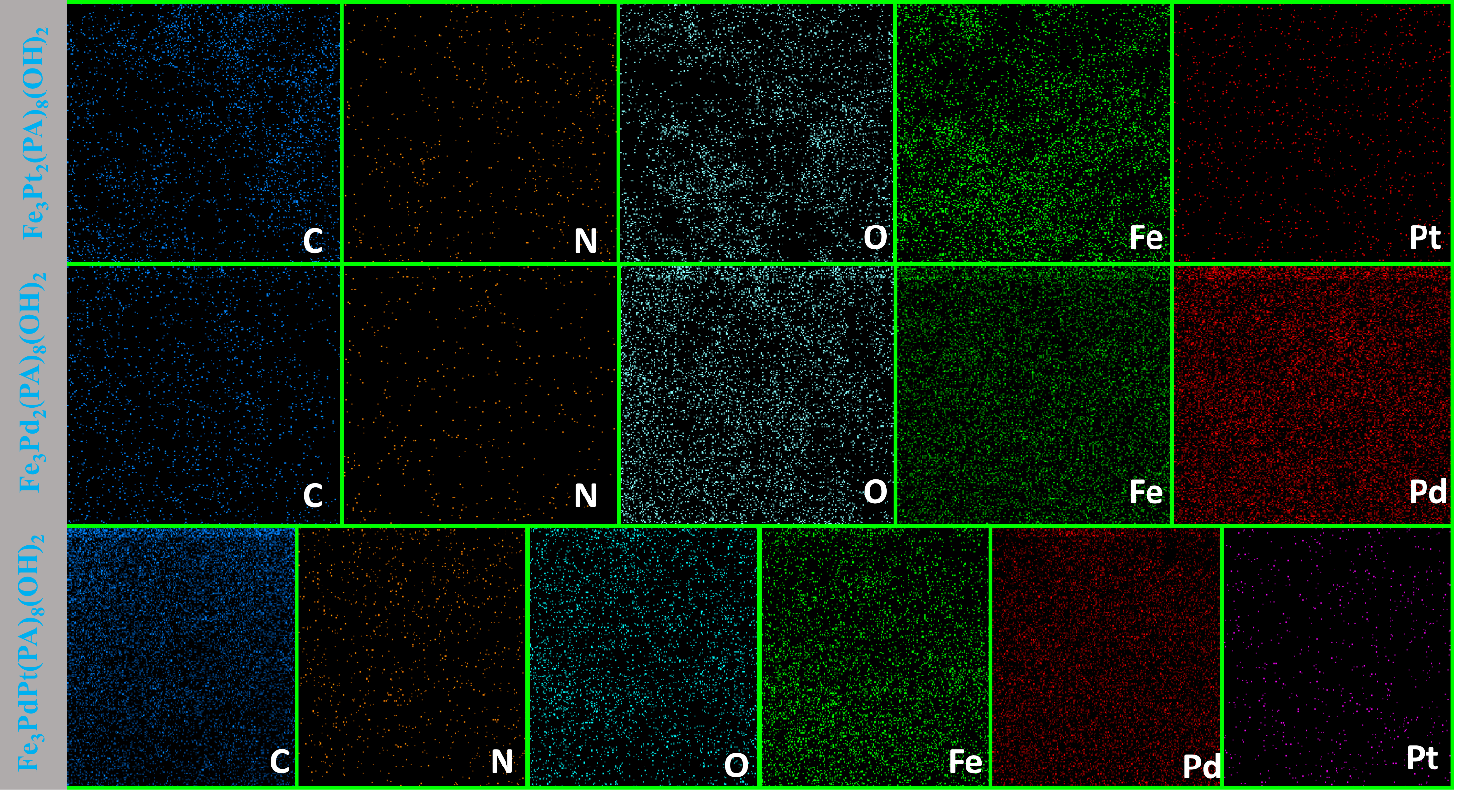


Figure S2 Elemental mapping images of Fe_3_Pt_2_[PA]_8_(OH)_2_(H_2_O)_4_, Fe_3_Pd_2_[PA]_8_(OH)_2_(H_2_O)_4_, and Fe_3_PtPd[PA]_8_(OH)_2_(H_2_O)_4_.

Table S2 Results from element chemical analysis and formulae of the sample prepared.

| Sample | Fe^a^ | Pt^a^ | Pd^a^ | C^a^ | O^a^ |
| --- | --- | --- | --- | --- | --- |
| Fe_3_Pt_2_[PA]_8_(OH)_2_(H_2_O)_4_ | 328.1 | 722.8 | - | 243.9 | 271.7 |
| Fe_3_Pd_2_[PA]_8_(OH)_2_(H_2_O)_4_ | 370.2 | - | 447.3 | 258.0 | 289.5 |
| Fe_3_PtPd[PA]_8_(OH)_2_(H_2_O)_4_ | 302.4 | 326.6 | 181.2 | 239.9 | 270.0 |

^a^ Mass percentage.

**
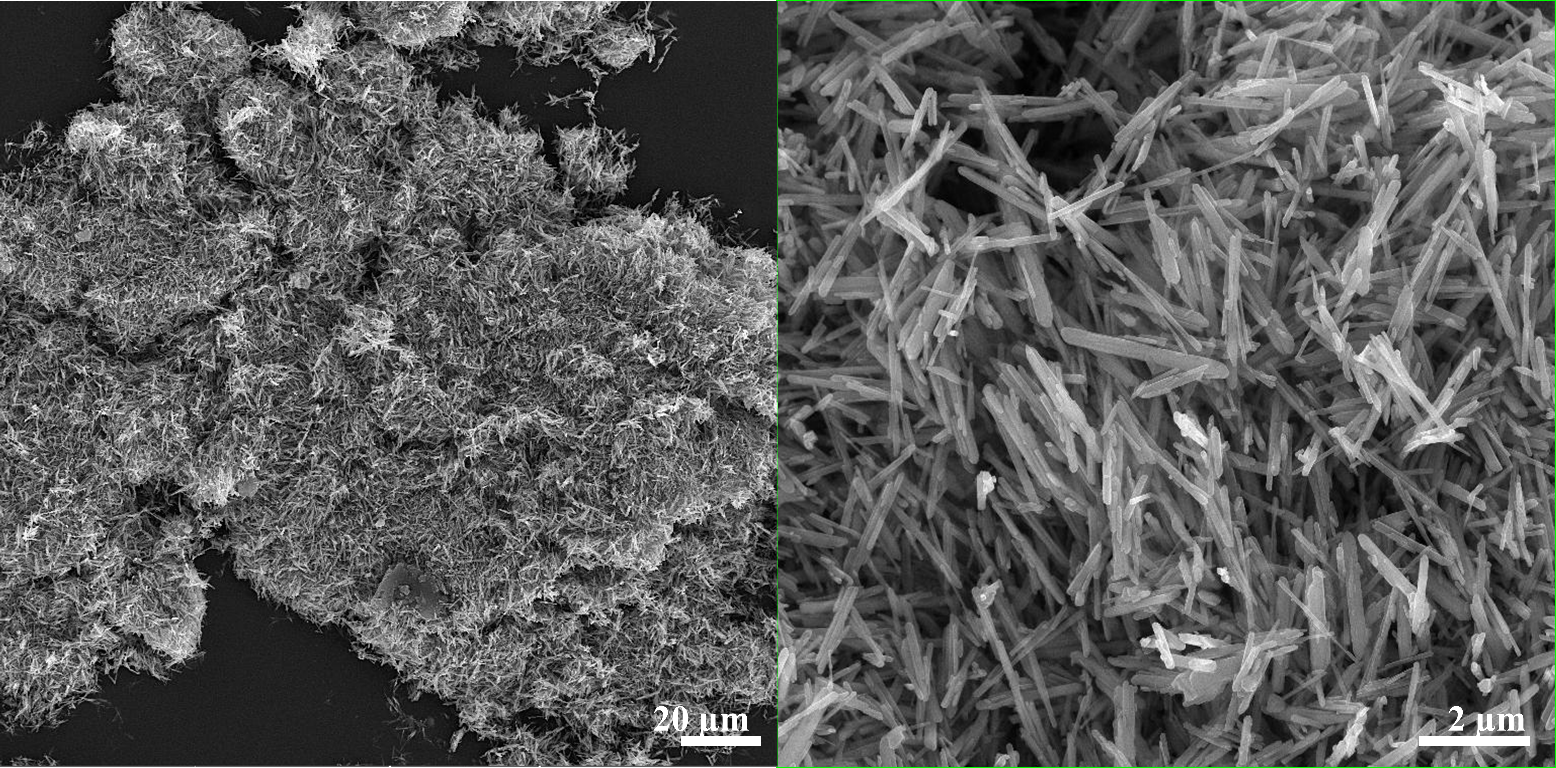
**

Figure S3 Low- and high- magnification FE-SEM images of the Fe_3_PtPd[PA]_8_(OH)_2_(H_2_O)_4_ hybrid nanorods

1. * Tel.: +98 21 82883417; Fax: +98 21 8288006544.

   E-mail address: [yyamini@modares.ac.ir](file:///C:\Users\shahram\Desktop\yyamini@modares.ac.ir) (Y. Yamini). [↑](#footnote-ref-1)
